# Expectant management of placenta accreta after a mid-trimester pregnancy loss: a case report and a short review

**DOI:** 10.1515/crpm-2021-0008

**Published:** 2022-02-01

**Authors:** Diletta Fumagalli, Tommaso Bignardi, Angelo Vanzulli, Paola Francesca Corbella, Mario Giuseppe Meroni, Maria Lieta Interdonato

**Affiliations:** Department School of Medicine and Surgery, Obstetrics and Gynecology Branch, University of Milano-Bicocca, Milan, Italy; Department of Obstetrics and Gynaecology, Niguarda Ca’ Granda Hospital, Milan, Italy; Department of Diagnostic and Interventional Radiology, Niguarda Ca’ Granda Hospital, Milan, Italy

**Keywords:** expectant management, mid-trimester, placenta accreta, placenta accreta spectrum

## Abstract

**Objectives:**

Placenta accreta spectrum (PAS) disorders are a significant cause of maternal morbidity and mortality. Traditionally women with PAS are offered surgery, while expectant management is still considered investigational.

**Case presentation:**

We present a case of expectant management of PAS after pregnancy loss at 19-weeks. PAS was suspected at sonography and confirmed by MRI. Patient was offered expectant management to preserve fertility. This consisted of leaving the placenta *in situ*, followed by in- and out-patient clinical and sonographic examinations and blood tests. After five weeks placental detachment occurred without major complications.

**Conclusions:**

Our report suggests that expectant management could be a safe option in selected cases of PAS after mid-trimester pregnancy loss. We recommend expectant management should be offered in referral centers for PAS.

## Introduction

Placenta accreta spectrum (PAS) disorders have been increasingly reported in the last four decades. PAS can be a cause of massive maternal hemorrhage and represents a significant cause of maternal morbidity and mortality. The management of this condition is complex and usually requires a multidisciplinary approach involving interventional radiologists and gynecologic oncology surgeons. Furthermore access to a blood bank capable of employing massive transfusion protocols should be available.

Early diagnosis of PAS is essential to improve patient outcomes. Transvaginal or transabdominal sonography (TVS, TAS) by an expert is the technique of choice for the diagnosis [1]. TVS allows an earlier recognition of PAS even in the first or second trimester, before major complications of placental invasion may arise.

Traditionally women with PAS have been offered surgical management, either with hysterotomy of hysterectomy at the time of caesarean delivery, however other conservative options have been advocated to achieve uterine preservation and avoid hemorrhage [2]. Expectant management by leaving the placenta *in situ* may be an alternative option for women wishing to preserve fertility; however, literature reports about women with PSA managed expectantly after a mid-trimester pregnancy loss/termination are scant. We present a successful case of expectant management of retained placenta accreta that was diagnosed after a mid-trimester pregnancy loss.

## Case presentation

A 38-year-old nulliparous woman was referred to our tertiary care center after a pregnancy loss at 19-weeks of gestation. The fetus was expelled at patient’s home. The woman was brought by ambulance to a primary care hospital about 30 min after delivery. She was diagnosed with placental retention, and PAS was suspected after an attempt of manual evacuation failed. Vital signs were normal on arrival and during the attempt of manual evacuation; the estimated blood loss was 200 mL. Patient’s medical history was significant for obesity (BMI 31 kg/m^2^), a previous laparotomic myomectomy for multiple uterine fibroids, and a recent diagnosis of recurrent multiple uterine fibroids. She had conceived through intracytoplasmic sperm injection (ICSI).

**Figure 1:**
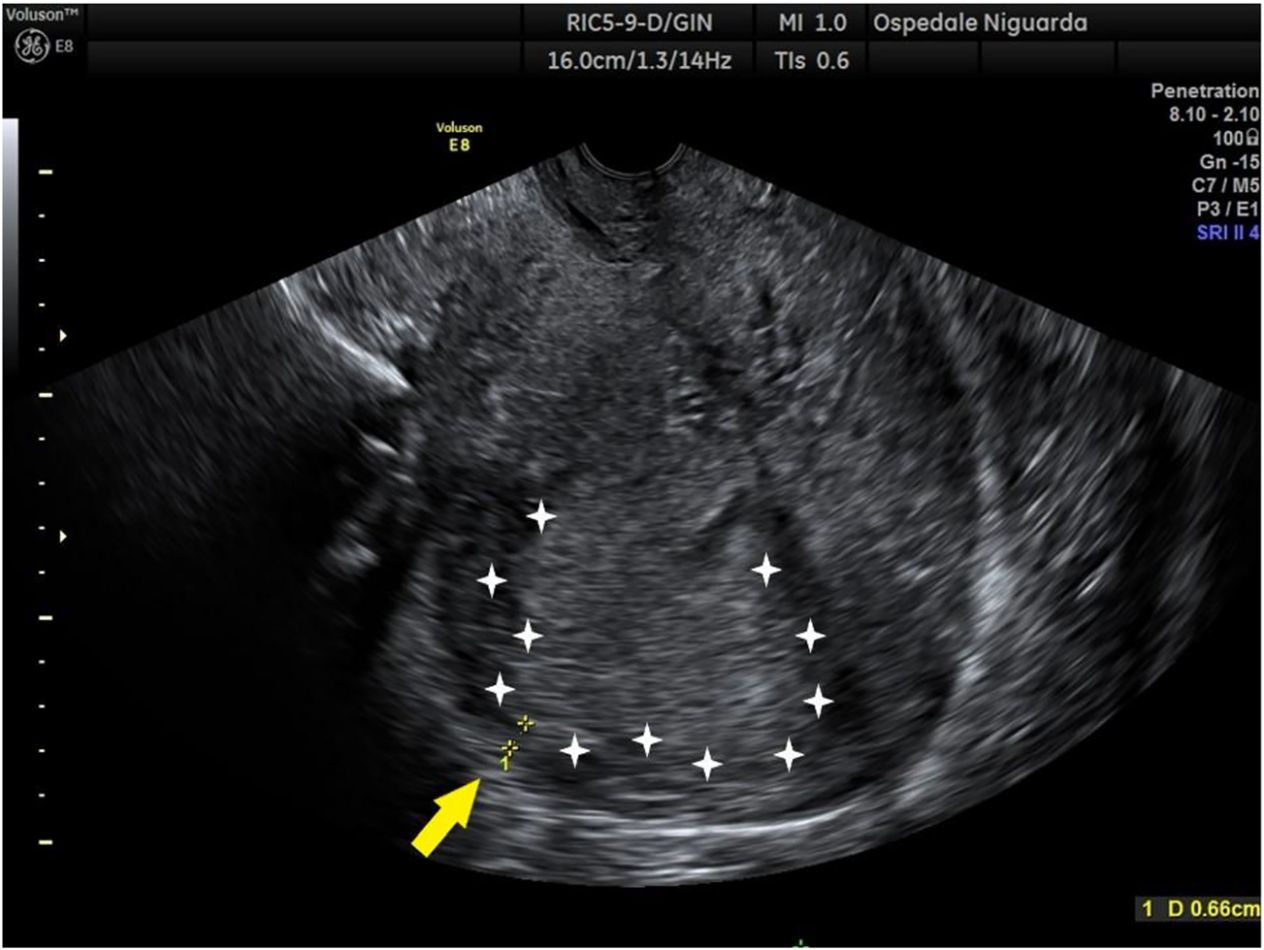
Grayscale TVS after pregnancy loss at 19 weeks, showing placenta accreta with irregular margins (white calipers), loss of the retroplacental clear space, myometrial thinning (arrow).

**Figure 2:**
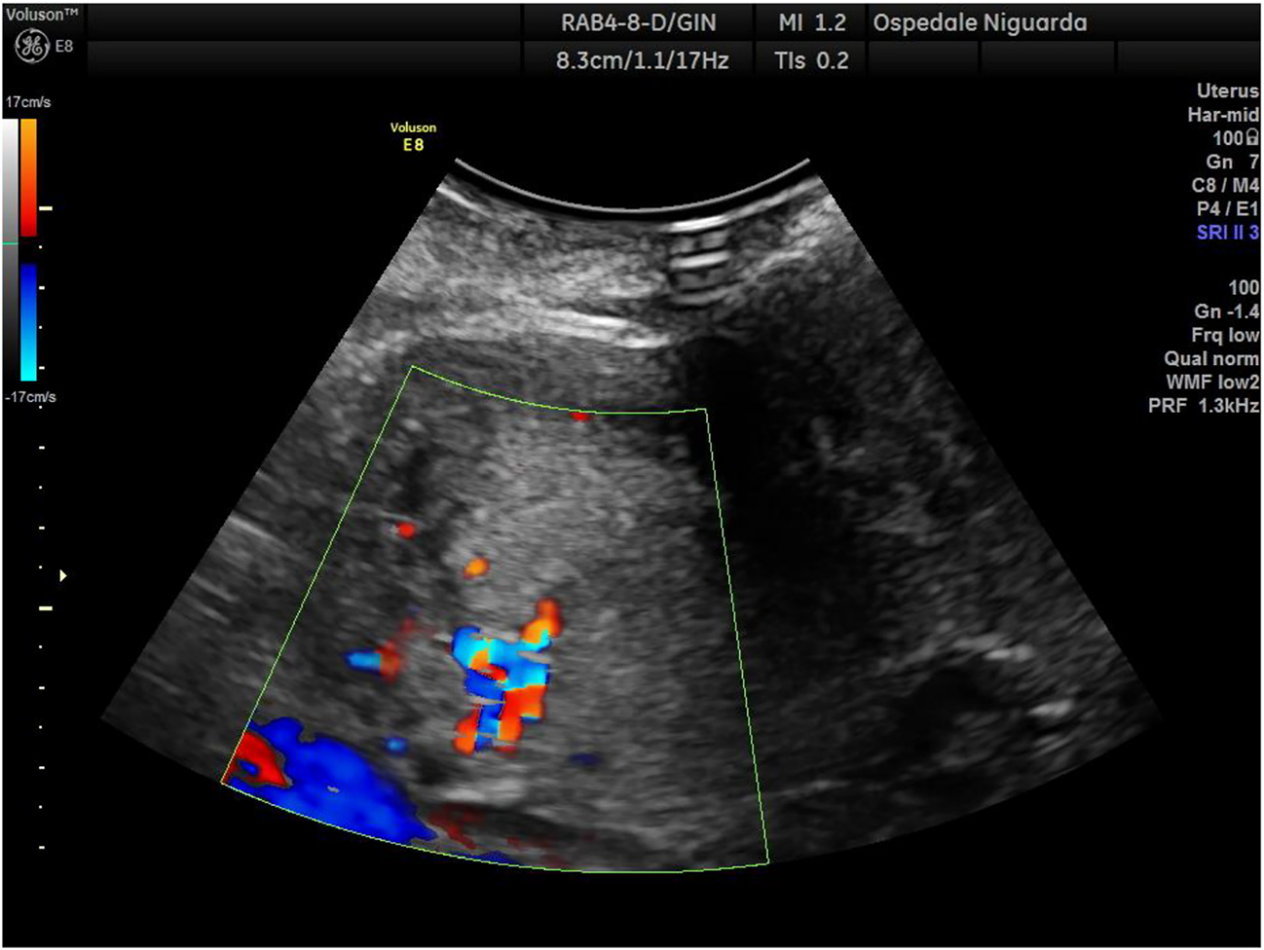
Color-Doppler TVS showing increased vascularization at the placenta-myometrium interface.

**Figure 3:**
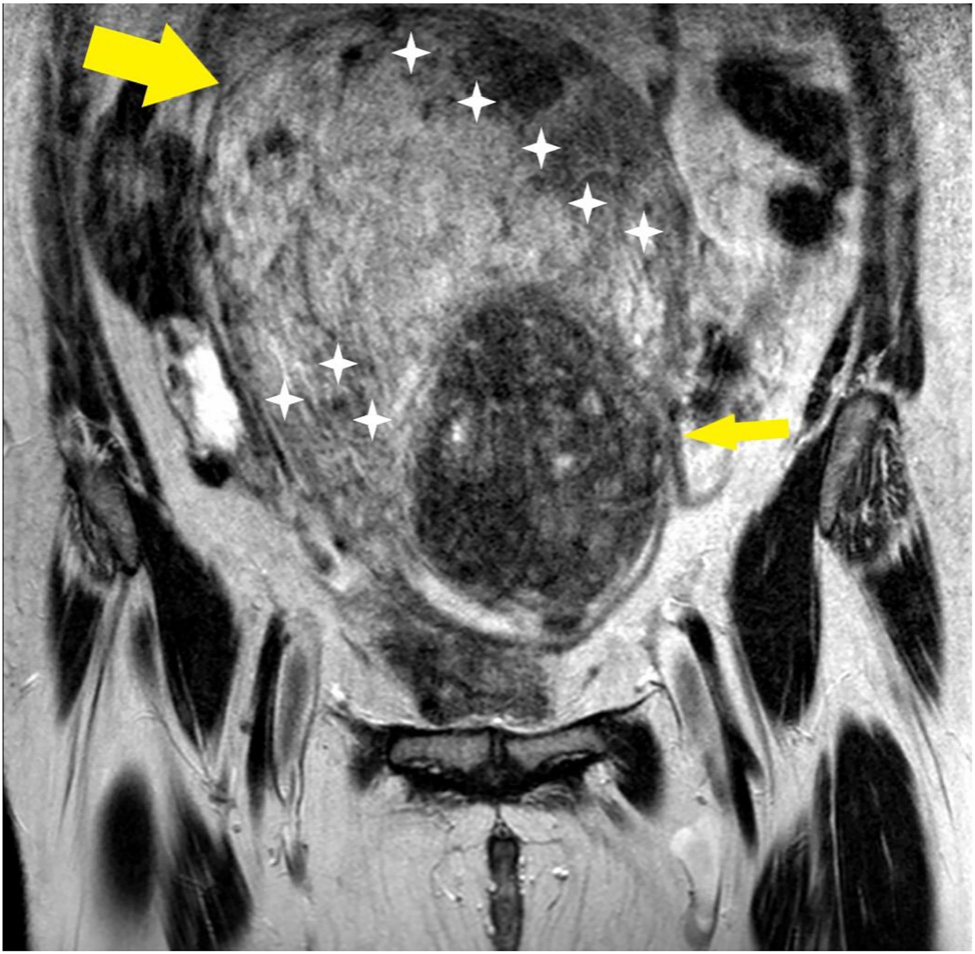
Contrast-enhanced MRI. Coronal T2 weighed acquisition: the uterus is enlarged and shows a hypointense fibroma on the left side (thin arrow). The placenta (delimited by white calipers) is implanted on the right side and marked thinning of the myometrium is evident (large arrow).

**Figure 4:**
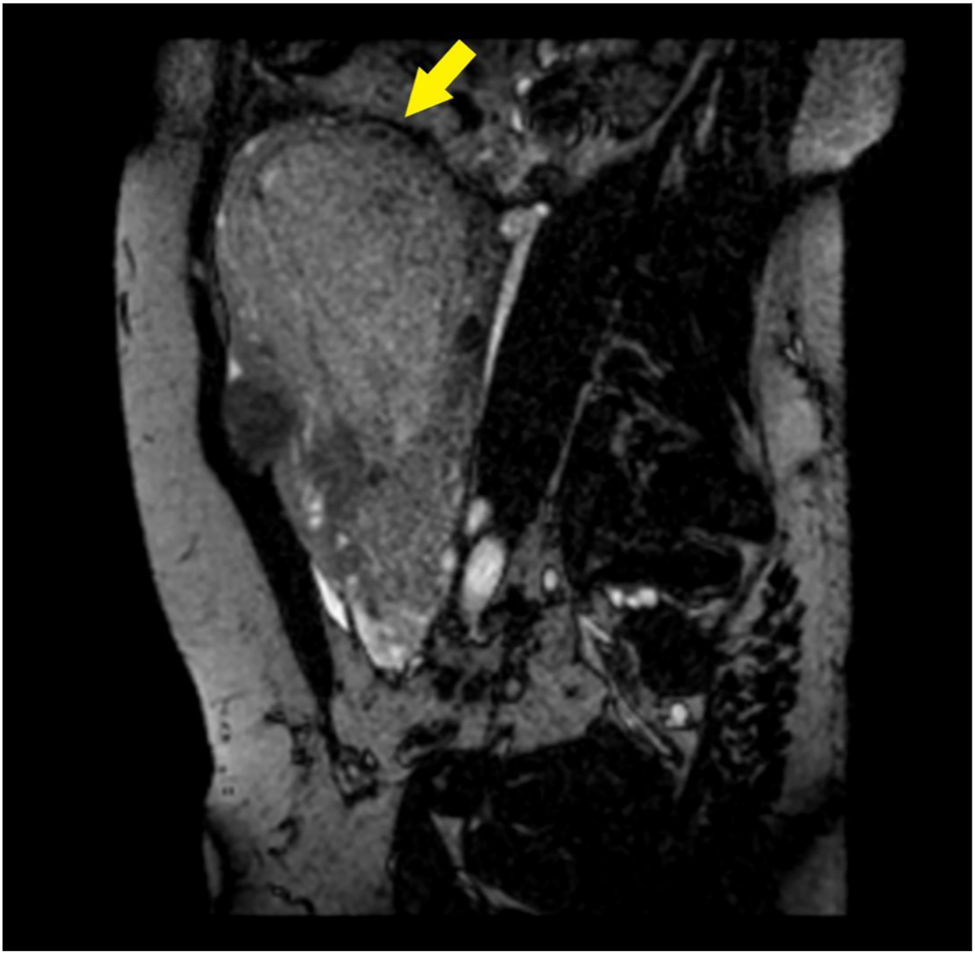
Contrast-enhanced MRI. Para-sagittal BFFE acquisition: the placenta causes marked thinning of the myometrium (arrow).

The patient was transferred by ambulance to our tertiary care center after 2 h from delivery; normal vital signs were registered during transfer and upon arrival at our emergency department, and vaginal bleeding was absent. A Foley catheter was placed with an output of 300 mL of clear urine. She was started on antibiotic prophylaxis with amoxicillin/clavulanate 875/125 mg i.v. q8hr because of mild leukocytosis and C-reactive protein (CRP) elevation (Table 1). TVS and TAS showed an increased uterine size, with multiple (>4) fibroids with the largest diameter of 120 mm, and a retained placenta. A thorough sonographic examination of the placenta was difficult because of the presence of uterine fibroids, obesity, and a posterior placenta. However, we suspected an area of myometrial invasion in the posterior uterine wall, where the placenta showed irregular margins, loss of the retroplacental clear space, myometrial thinning, and increased color doppler signal (Figures 1 and 2, Supplementary Material, Videoclip 1 [Grayscale TVS showing a posterior area of placental accretism]). To confirm the diagnosis, we asked for gadolinium-enhanced MRI. T1- and T2-weighted scans showed a diffusely enlarged uterus, with pregnancy-induced myometrial hypertrophy and irregular uterine contours, due to the presence of both intramural and subserous myomas. MRI confirmed the placental invasion of the myometrium in a posterior area extending about 60 mm, apparently without serosal infiltration (Figures 3 and 4).

**Videoclip 1:** Grayscale TVS showing a posterior area of placental accretism.

**Table 1:** Patient’s vitals and labs at presentation.

	1st admission	2nd admission
Blood pressure	126/72 mmHg	110/71 mmHg
Heart rate	98 bpm	90 bpm
Temperature	36.6 °C	36.2 °C
SatO_2_	96%	96%
Hb	9.9 g/dL	10.8 g/dL
WBC	12.33 × 10^9^/L	6.48 × 10^9^/L
CRP	8.5 mg/L	0.4 mg/L
Fibrinogen	340 mg/dL	310 mg/dL
INR	1.08	1.07
aPTT ratio	1.01	1.02

After counselling the woman opted for expectant management and informed consent was obtained. She was monitored eight days as an in-patient, undergoing daily monitoring of blood tests (complete blood count [CBC], CRP, fibrinogen, prothrombin time [PT], partial prothrombin time [PTT]) and vitals and sonography. The hospital stay was uneventful, and the patient was discharged and followed-up weekly with: clinical assessment focused on signs and symptoms of pelvic infection and symptoms of PV bleeding; both grayscale and doppler sonographic assessment of the placenta, and uterine artery (UA) doppler indices; blood tests. Patient was instructed to come back to our emergency department in case of bleeding, vaginal discharge, fever, or pelvic pain, and to avoid travel. Interventional radiologists and gynecologic oncology surgeons at our institution were asked to review the case and arrangements were made in case emergency uterine arteries embolization (UAE) or surgery would be needed.

The outpatient follow-up was unremarkable, and the blood tests returned to normal after two weeks. At color-Doppler TVS the placental tissue showed loss of arterial vascularization after four weeks, whilst UA doppler velocimetry was still showing a pregnancy-like low-resistance flow (mean PI 1.01, RI 0.61). After five weeks the patient was brought by ambulance to our emergency department because of heavy PV bleeding, with an estimated blood loss of 250 mL. Upon presentation vital signs and blood tests were normal (Table 1). It was not possible to collect placental tissue on admission; the placenta had probably been expelled at home. Patient was treated with i.v. tranexamic acid and fluids for three days, until the bleeding subsided spontaneously. TVS showed absence of placental tissue inside the uterus. UA doppler went back to a non-pregnancy, high-resistance flow. The following hospital stay was uneventful, and the patient was discharged seven days later.

## Discussion

This case report suggests that PAS disorders may be managed expectantly, by leaving the placenta *in situ*, in women wishing to preserve fertility. In these cases, patient’s counselling must consider hemorrhagic and infectious risks. In case of clinical stability, these women could be offered a strict and cautious follow-up as outpatients, but they should be referred to a tertiary center if any complication arise, because they may need advanced hemostatic techniques (i.e., UAE) or complex surgery by a gynecology oncologist.

Placenta accreta is defined as abnormal trophoblast invasion of part or all the placenta into the myometrium of the uterine wall. PAS refers to the range of pathologic adherence of the placenta, including placenta accreta – when the villi adhere to the myometrium, placenta increta – when the villi invade the myometrium, and placenta percreta, which includes villous invasion of the whole thickness of the myometrium up to uterine serosa, and sometimes adjacent pelvic organs [3]. It has been postulated that any primary uterine anomaly or secondary damage to the uterus can lead to PAS [4]. The incidence of this condition has been progressively increasing during the last four decades, probably due to an increase in risk factors – strong epidemiologic data have linked PAS disorders directly to an increase in cesarean delivery rates [5], as uterine scars may cause a localized failure of decidualization [6]. Other gynecologic procedures that result in uterine scarring have been associated with PAS, as uterine curettage, manual delivery of the placenta, hysteroscopic surgery, endometrial ablation [7]. PAS disorders were also reported in women with uterine pathologies, such as bicornuate uterus, adenomyosis, fibroids, however the high prevalence of these conditions in the general population and the lack of evidence of their association with PAS suggest they are not a major risk factor for PAS development [8]. Assisted reproductive technologies (ART) have been linked to an increased risk of PAS by several studies. Recently, a retrospective study by Salmanian et al. [7] found that *in vitro* fertilization is an independent risk factor for PAS disorders, even though the association is less considerable than other major risk factors as previous cesarean delivery; different types of *in vitro* fertilization (cryopreserved vs. fresh embryo transfer), uterine preparation and protocols of ovarian stimulation may result in different risks of PAS. Cryopreserved embryo transfer seems to have the highest odds of PAS when compared to other techniques, but the exact pathogenetic mechanism remains unknown [8, 9].

TVS and TAS are the techniques of choice for the diagnosis of PAS, with an overall excellent performance [1, 12]. Standardized descriptors for ultrasound reports have been proposed both for grayscale and Doppler imaging in women at high risk for PAS [10, 11]: placental lacunae, loss of the retroplacental hypoechoic zone between the placenta and myometrium, thinning of retroplacental myometrium, abnormal uterine contour (placental bulge), exophytic masses or bridging vessels beyond the uterine serosa. However, these markers have been studied in a high-risk population, with previous cesarean sections or placenta previa. Their presence and significance in a low-risk population remains largely unknown [11].

Moreover, prenatal diagnosis of PAS with ultrasonography has traditionally focused on the second and third trimester of pregnancy. First trimester screening of PAS among women with a previous cesarean section has been proposed at the time of NT scan (11–14 weeks of gestation) [12]; cases of PAS in patients with a previous cesarean delivery may start as cesarean scar pregnancies, that can be diagnosed in the first trimester. Early pregnancy markers for cesarean scar pregnancy are gestational sac implantation in part or totally within the cesarean scar; the gestational sac may have a teardrop or triangular shape. A low implantation pregnancy may also be diagnosed: a gestational sac located close to the internal cervical os (up to 8 6/7 weeks of gestation) and/or placental implantation located posterior to a partially filled maternal bladder (up to 13 6/7 weeks of gestation) [11]. Regarding our case report, an earlier diagnosis of PAS may have modified pregnancy management and patient’s counseling and decisions about the continuation of the pregnancy itself; the patient, however, did not have major risk factors for PAS, and according to current knowledge would not have been included in a first-trimester screening program for PAS. Future research may evaluate the role of first-trimester screening for PAS not only for patients with major but also with minor risk factors.

MRI has been increasingly used as an adjunct to ultrasound in the presurgical evaluation of topography and depth of placental invasion. A consensus statement that aims for uniformity in MRI acquisition, interpretation and reporting of PAS has been recently published [13].

With regards to treatment options, PAS disorders have been traditionally managed with surgery, either conservative or demolitive [14, 15]. Other therapeutic options have been advocated to preserve fertility and avoid hemorrhage or injury to other pelvic organs. Expectant management, defined as leaving the placenta either partially or totally *in situ*, is still considered an investigational approach [16]. With expectant management the cord is ligated near the placenta and the entire placenta is left *in situ*, or only the placenta that spontaneously separates is removed before uterine closure. This approach is based on the following evidence-based clinical concepts: cesarean hysterectomy is considered the gold standard treatment for invasive PAS, but it is associated with high rates of severe maternal morbidity; the attempt of manual removal is associated with severe maternal morbidity because it leaves placental tissues connected to myometrium and to large feeding vessels, which can be responsible for uncontrolled massive hemorrhage [17]. Expectant management leads to a progressive decrease in blood circulation within the uterus, parametrium and placenta: this will result in villous tissue secondary necrosis. The placenta should spontaneously detach itself from the uterus [17]. In a multicenter observational study by Sentilhes et al., 22% of women with PAS required hysterectomy after an attempt of expectant management. Those with successful expectant management (78%) had a median time to placental involution of 13.5 weeks [2]. There are limited data on the conservative management of placenta percreta; when managed conservatively hysterectomy can be avoided in up to 60% of cases, but 40% of them had major complications [18]. One recent study by Biele et al. stated that a rare, but serious complication of conservative management of placenta percreta is disseminated intravascular coagulation (DIC) [17]; this condition has been reported even in previous studies [18]. The hemostatic changes and the coagulation state in PAS disorders are unknown both before delivery and during conservative or expectant management, but it has been postulated that the invasive growth of the placenta may lead to increased local and systemic coagulation activity, up to early consumption of coagulation factors, with a fibrinogen decrease and a d-dimere increase; consequently, a secondary postpartum hemorrhage or a DIC may follow. Biele et al. suggest that expectant management follow-up should include coagulation parameters for early diagnosis of DIC and, in case of altered values of fibrinogen and d-dimer, oral dosage of tranexamic acid may be considered as therapeutic option.

In 2019 the International Society for Abnormally Invasive Placenta (IS-AIP) [19] stated that, as expectant management appears to be associated with less blood loss and lower transfusion requirements than both hysterectomy and uterus-conserving surgery, and successful for 60–93% of patients, this can be offered as an appropriate management strategy for women wishing to preserve their fertility and in cases when hysterectomy is at high risk of surgical complications. High-quality evidence from large, multicentric, randomized studies is, however, still needed.

Fertility seems to be spared after expectant management, although current data are limited. Women trying to conceive again after PAS should be adequately counseled about the risk of recurrence, which is reported to be around 30% [17, 18].

Overall reports in the literature about expectant management of PAS are still scant and usually focusing on hysterectomy vs. conservative management in the third trimester. We found only one study by Ou et al. describing the outcomes of expectant or conservative management of women with PAS who had pregnancy losses or terminations in the mid-trimester; authors achieved uterine preservation in all of 28 cases who were managed expectantly with or without the use of adjuvant treatments (UAE, MTX, mifepristone) [20]. Some Authors have advocated the use of adjuvant therapies as methotrexate or prophylactic uterine artery embolization for conservative or expectant management of PAS. The IS-AIP does not recommend methotrexate use [19], as there is no evidence of benefit, while there is evidence from Sentilhes et al. [2] for potential significant harm including maternal mortality. No evidence of benefit has been found for prophylactic uterine artery embolization, too. Therapeutic embolization for postpartum hemorrhage may avoid hysterectomy in conservatively or expectantly managed women [19].

In our case we have administered antibiotic therapy because we suspected an initial infection. Prophylactic antibiotic therapy may be included in the standard care of expectant management, as in previous studies infections were reported in 28.1% of cases, with an incidence of sepsis of 4.2%. One case of septic shock leading to maternal death has also been described [2].

For monitoring the degree of placental involution, we have used color-Doppler and UA doppler velocimetry. Our findings suggest that a shift from a low- to a high-resistance flow, along with the loss of placental vascularization at color-Doppler, may provide reassurance about a correct involution of the placental site.

## Take home message

Our case report suggests that expectant management may be a safe option after a mid-trimester pregnancy loss; managing women with PAS expectantly in the mid-trimester could provide better outcome compared to the third trimester, because spontaneous detachment of placental tissue may occur earlier and with a lower risk of hemorrhage. We believe that expectant management may be offered to all women with a PAS after a mid-trimester pregnancy loss who are deemed as clinically stable.

We recommend that expectant management comprises a close follow-up (at least weekly) with a full clinical evaluation, sonography, and blood tests (CBC, fibrinogen, PT, PTT, CRP); the patient must be adequately counselled about the potential benefits of expectant management, like the preservation of fertility and avoidance of surgery, and about risks, in particular infection, sepsis, DIC, and possible need of UAE or demolitive surgery, which may be needed in 20–40% of cases after an attempt of expectant management. Patients must be informed about possible warning signs of the conditions listed above. In case of signs or symptoms of infection or bleeding, a timely readmission of the patient should be considered. Expectant management must be abandoned if the patient is not clinically stable because of massive hemorrhage, or if impending DIC or sepsis are suspected based on clinical and laboratory findings.

In conclusion, we believe that expectant management is a reasonable option after mid-trimester pregnancy loss with PAS, however prospective data are needed to assess safety and outcomes of this kind of management. In our opinion, a multidisciplinary approach involving interventional radiologists, gynaecologic oncology surgeons and obstetrical anaesthesiologists is the way to improve patient outcomes.

## Supplementary Material

Supplementary Material Details
