# Supplementary material for: Expectant management of placenta accreta after a mid-trimester pregnancy loss: a case report and a short review
Source: Case Rep Perinat Med. 2022 Feb 1;11(1):20210008. doi: 10.1515/crpm-2021-0008 (PMC11800657; doi:10.1515/crpm-2021-0008)
Supplement: Supplementary file 3 — Supplementary Material Details [file j_crpm-2021-0008_suppl.docx]

Videoclip 1. Grayscale TVS showing a posterior area of placental accretism.
